# Universal Approach to Direct Spatiotemporal Dynamic In Situ Optical Visualization of On‐Catalyst Water Splitting Electrochemical Processes

**DOI:** 10.1002/advs.202401258

**Published:** 2024-04-22

**Authors:** Gaurav Bahuguna, Fernando Patolsky

**Affiliations:** ^1^ School of Chemistry Faculty of Exact Sciences Tel Aviv University Tel Aviv 69978 Israel; ^2^ Department of Materials Science and Engineering the Iby and Aladar Fleischman Faculty of Engineering Tel Aviv University Tel Aviv 69978 Israel

**Keywords:** confocal microscopy, HPTS, in‐operando analysis, spatiotemporal dynamic analysis, visualizing water splitting

## Abstract

Electrochemical reactions are the unrivaled backbone of next‐generation energy storage, energy conversion, and healthcare devices. However, the real‐time visualization of electrochemical reactions remains the bottleneck for fully exploiting their intrinsic potential. Herein, for the first time, a universal approach to direct spatiotemporal‐dynamic in situ optical visualization of pH‐based as well as specific byproduct‐based electrochemical reactions is performed. As a highly relevant and impactful example, in‐operando optical visualization of on‐catalyst water splitting processes is performed in neutral water/seawater. HPTS (8‐hydroxypyrene‐1,3,6‐trisulfonicacid), known for its exceptional optical capability of detecting even the tiniest pH changes allows the unprecedented “spatiotemporal” real‐time visualization at the electrodes. As a result, it is unprecedentedly revealed that at a critical cathode‐to‐anode distance, the bulk‐electrolyte “self‐neutralization” phenomenon can be achieved during the water splitting process, leading to the practical realization of enhanced additive‐free neutral water splitting. Furthermore, it is experimentally unveiled that at increasing electrolyte flow rates, a swift and severe inhibition of the concomitantly forming acidic and basic ‘fronts’, developed at anode and cathode compartments are observed, thus acting as a “buffering” mechanism. To demonstrate the universal applicability of this elegant strategy which is not limited to pH changes, the technique is extended to visualization of hypochlorite/ chlorine at the anode during electrolysis of sea water using N‐(4‐butanoic acid) dansylsulfonamide (BADS). Thus, a unique experimental tool that allows real‐time spatiotemporal visualization and simultaneous mechanistic investigation of complex electrochemical processes is developed that can be universally extended to various fields of research.

## Graphical abstract

An unique experimental tool that allows real‐time spatiotemporal visualization and simultaneous mechanistic investigation of complex electrochemical processes is developed, that can be universally extended to various fields of research. As an impactful example, in‐operando visualization of water splitting reactions under unbuffered neutral and seawater conditions is performed which unveils unprecedented insights on the electrocatalytic reactions.

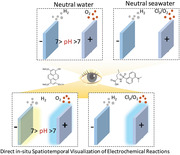

## 1 Introduction

Electrochemistry is one of the most important domains of science and is known since decades for its immense applicability in various field of interest. It has a widespread application in the present day most important areas of energy^[^ ^1^, ^2^ ^]^ and healthcare.^[^ ^1^, ^3^, ^4^, ^5^, ^6^ ^]^ As a result, this high‐impact research field has since its discovery received enormous research attention and efforts, with continuous accomplishment of fundamental and technological developments.^[^ ^7^, ^8^, ^9^ ^]^ In spite of these significant research achievements, the field of electrochemistry is still far from fulfilling its intrinsic potential, with the scope of still accomplishing tremendous future developments. These developments can lead to the evolution of next‐generation energy storage, energy conversion, and healthcare devices for a sustainable and healthy development of mankind.^[^ ^8^, ^9^, ^10^, ^11^ ^]^ The comprehensive applicability of electrochemistry, and electrochemical reactions, is still restricted due to the current limited understanding, and lack of experimental tools for the in‐depth characterization, of various electrochemical processes of interest. Thus, the discovery and development of innovative scientific and technological avenues toward the understanding of complex electrochemical processes is highly desired and required.^[^ ^12^, ^13^, ^14^ ^]^ Thus, the in situ spatiotemporal “visualization” of electrochemical processes can represent a major breakthrough in this field of research and may lead to unveiling yet unexposed fascinating insights in the field.^[^ ^15^ ^]^ However, it is unfortunate that despite major advancements in the development of optical microscopic techniques, the spatiotemporal dynamic ‘visualization’ of electrochemical reactions has not yet been demonstrated.

Fluorescence‐based imaging techniques represent one of the central routes for achieving the goal of spatiotemporal visualization of electrochemical reactions.^[^ ^16^, ^17^, ^18^ ^]^ Few reports are available on the sensing of electrochemical reactions using a fluorophore that is responsive to any one of the reaction products.^[^ ^19^ ^]^ This, however, requires the application of highly specific fluorophores for the specific product, and thus cannot be extended universally to different electrochemical processes.^[^ ^19^ ^]^ Additionally, most of the works in the literature report on the fluorescence sensing of the electrochemical products, rather than the in situ imaging or visualization of any electrochemical reactions.^[^ ^17^, ^19^ ^]^ Furthermore, it is very important to provide the quantitative in‐operando visualization of the electrochemical reactions, which is usually missing in existing literature investigations.^[^ ^20^ ^]^ Acid/base (or pH) fluorescent probes can be one of the best candidates for visualization purposes, as most of the electrochemical reactions involve the oxidation or reduction of specific moieties which cause a concomitant decrease or increase in the environment pH, at one or both of the electrodes simultaneously.^[^ ^21^, ^22^ ^]^ These changes in pH can be monitored using acid/base (pH)‐sensitive fluorescent probes for monitoring the in situ progress of any electrochemical reaction. The pH‐based visualization of electrochemical reactions can be universally applied to various electrochemical processes, however presently suffers majorly due to a limited range of suitable fluorescent probes displaying chemical and electrochemical stability in the voltage window of interest, the use of multiple fluorescent dyes for specific tasks and voltage range of interest, the lack of a suitable visualization approach, etc. which overall make the spatiotemporal visualization process very complex. Recently, Roberts et al.^[^ ^23^ ^]^ used a combination of dyes to monitor an electrocoagulation process by observing the pH changes in the range of 1.5–8.5. To address the issues, we have explored HPTS (8‐hydroxypyrene‐1,3,6‐trisulfonicacid) as a fluorescent probe, with a neutral pKa of 7.4 in the ground state. HPTS is a well‐known photoacid molecular species, displaying a sharp transition from a pKa of 7.4 in the ground state, being a weak acid, to a pKa of ≈0.5 in its photo‐excited state, making it a strong acid upon excitation. The exceptional properties of the HPTS molecule allow it to monitor any minute changes from neutrality to acidic or basic conditions, thus allowing the use of a single fluorescent probe. Furthermore, the optical fluorescence properties of the molecule allow the ratiometric sensing of the acid/basic transitions and thus can be used for the reliable qualitative and quantitative analysis of any small changes in the environment.^[^ ^11^, ^16^, ^24^, ^25^ ^]^

In the past few decades, electrocatalytic water splitting for hydrogen gas production was established as a basis for sustainable development and has led to significant progress in terms of efficiency and achievable low overpotentials under alkaline/acidic conditions.^[^ ^26^, ^27^, ^28^, ^29^, ^30^ ^]^ Corrosive environment, use of additives and expensive separators have very recently shifted the research attention to the realization of neutral water splitting.^[^ ^31^, ^32^, ^33^, ^34^, ^35^, ^36^, ^37^ ^]^ However, it is at an initial state and necessitates innovative characterization tools for a better understanding of the electrocatalytic water splitting under unbuffered neutral conditions.^[^ ^38^ ^]^ In the recent past, Zhang et al. have performed in situ high‐speed and micro visualization of electrochemical reactions by imaging the formation of gas bubbles in electrocatalytic reactions using a high‐speed camera.^[^ ^42^, ^43^, ^44^ ^]^ These in situ experiments provided insights on reaction interfaces and mass transport optimization by visualizing the gas bubbles during the water‐splitting reaction.^[^ ^45^, ^46^, ^47^ ^]^ However, it is to be realized that this “bubble‐based” visualization of water splitting reaction is limited due to the blind nature toward any chemical change happening in the electrochemical cell and thus necessitates innovative avenues that can visualize any chemical changes “even selectively” during any electrochemical reaction. In this work, for the first time, direct spatiotemporal dynamic in situ optical visualization of on‐catalyst water splitting processes is performed under neutral conditions by exploiting the exceptional fluorescent properties of the HPTS probe.^[^ ^38^ ^]^ Under neutral conditions, the oxidation and reduction of water (for the production of oxygen and hydrogen) at the anode and cathode, respectively, lead to the formation of acidic and basic environments around the electrodes at neutral conditions.^[^ ^39^, ^40^ ^]^ This associated formation of acid and base ‘fronts’ leads to a decrease and increase in the pH, respectively, which can be optically visualized directly by the opto‐electrochemical cell developed in this study.^[^ ^41^ ^]^ Recently, Obata et al.^[^ ^41^ ^]^ attempted bulk in situ imaging of the water splitting process under neutral conditions, and successfully observed the concomitant formation of acid and base and its associated pH changes. However, the work involved the use of a fluorescent strip in between the two electrodes, which led to limitations of minimum uncontrollable contact nature with the electrodes, resulting in contact aberrations, non‐microscopic imaging capabilities with low‐resolution and limited dimensional imaging, completely lacking 3D optical information of environmental chemical changes. Considering this broad research gap, the quest for a fluorescent probe is realized which can directly be added to the electrolyte medium for in‐operando visualization of water‐splitting reactions. Thus, an opto‐electrochemical cell, using HPTS as a fluorescent probe, was developed which demonstrates the following unprecedented advantages: 1) universal indicator for acid and base (pH) environment changes, 2) direct homogeneous addition into the cell electrolyte, 3) 3D spatial imaging capabilities, 4) ratiometric fluorescence analysis, and 5) easy qualitative and quantitative analysis in a single system. As a result, the opto‐electrochemical cell developed in this work is successfully able to quantitatively visualize the spatiotemporal development of acidic and basic fronts at the anode and cathode surfaces during the water‐splitting process reactions. Furthermore, the ability of the opto‐electrochemical cell to spatially visualize in situ water splitting reactions was practically applied to unveil further key insights on the efficiency of unbuffered neutral water splitting. Specifically, the possibility of ‘self‐neutralization’ via optimizing the distance gap between the electrodes, as well as the effect of applying different electrolyte “flow” conditions, at different flow rates, on the water splitting process were here investigated by in situ opto‐electrochemical imaging platform, under static and under‐flow electrolyte conditions, leading to the experimental discovery of key new insights that allow further enhancing the water splitting process under neutral conditions. Additionally, this approach was here universally applied for the spatiotemporal monitoring of chemical by‐products other than pH changes, such as the formation of hypochlorite and chlorine species using a specific fluorophore, concomitantly produced during the seawater splitting process under neutral conditions. This shows the universal nature of our novel experimental paradigm that can be extended to any electrochemical reaction per se.

## 2 Results and Discussion

**Figure 1:**
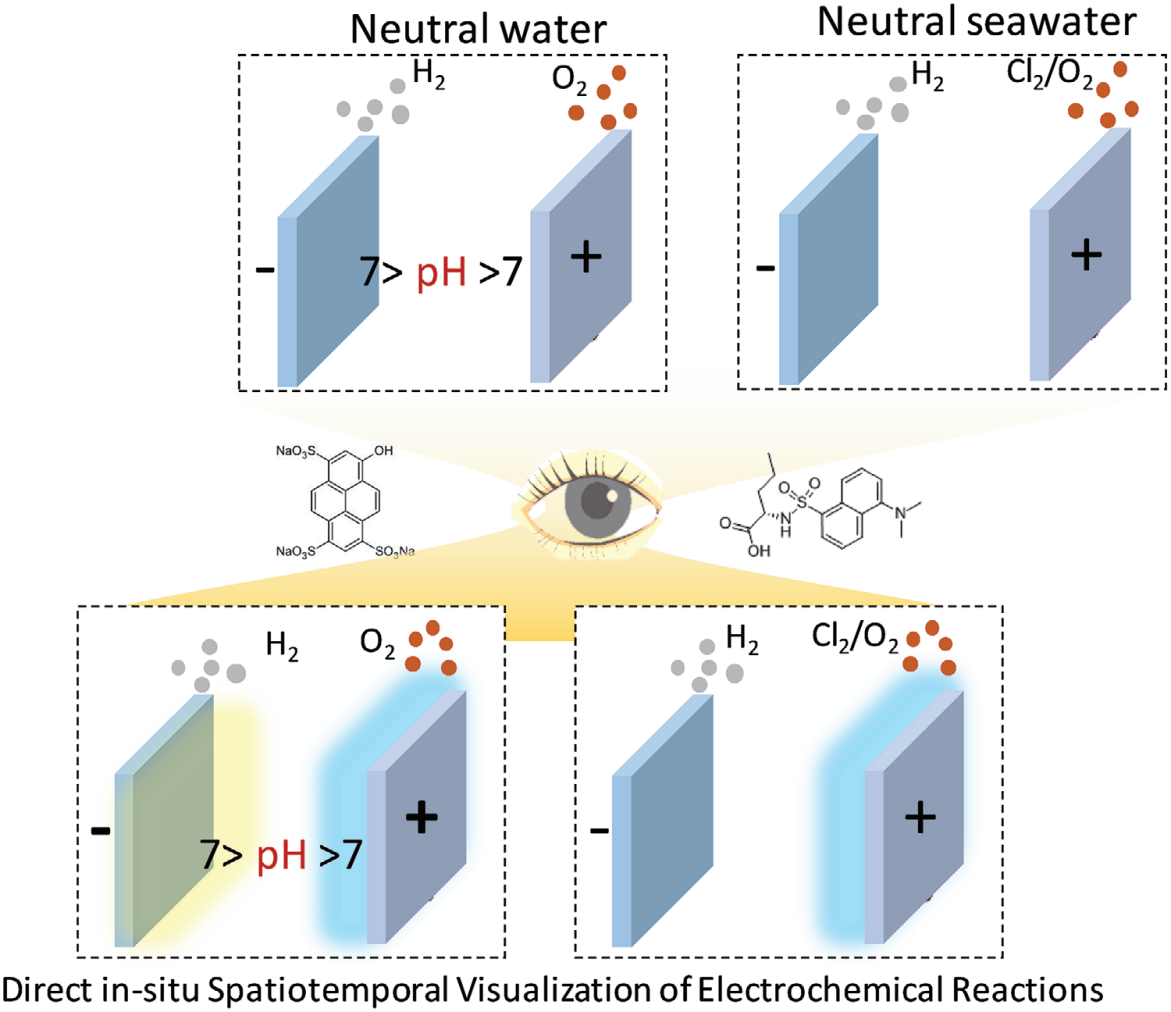
Schematic demonstration of the in situ optical spatiotemporal visualization of electrochemical reactions, considering the cases of neutral water and neutral sea water splitting as an example.

**Figure 2:**
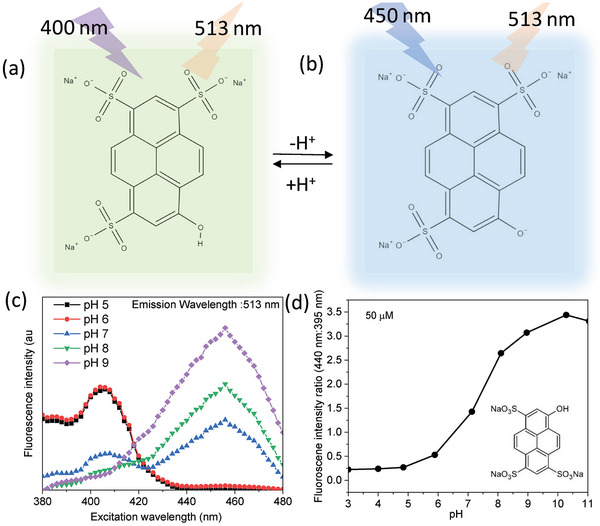
Chemical structure of the a) protonated and b) deprotonated forms of the fluorescent probe HPTS (8‐hydroxypyrene‐1,3,6‐trisulfonicacid). c) Fluorescence spectra (at 513 nm emission wavelength) of 50 µm HPTS solution at different pH (5–9) and d) Fluorescence intensity ratio (excitation ratio 440:395 nm) of HPTS solutions at different pH.

**Figure 3:**
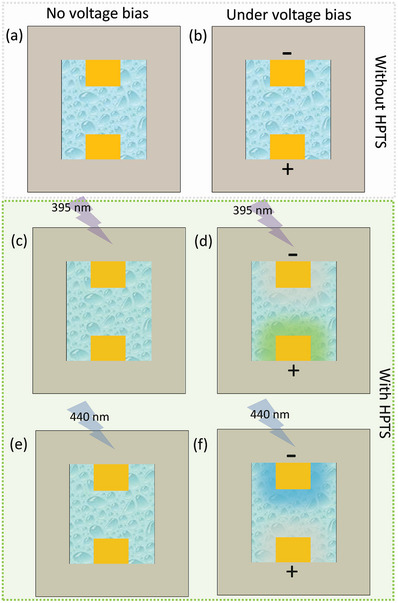
Schematic demonstration of the opto‐electrochemical spatiotemporal cell imaging approach (a,b) without HPTS probe, (c,d) under 395 nm and (e,f) under 440 nm excitation wavelengths.

**Figure 4:**
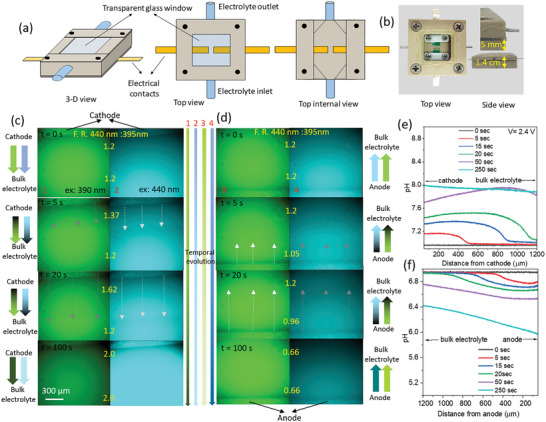
a) Schematic demonstration and b) digital photograph of the opto‐electrochemical cell used in this work. Spatiotemporal fluorescence imaging of the c) cathode and d) anode vicinity under electrochemical cell operation at 2.4 V, using 50 µm HPTS as fluorescent probe in 0.25 m Na_2_SO_4_ electrolyte. pH variations around e) cathode and f) anode vicinity as calculated via ratiometric analysis of (c) and (d).

**Figure 5:**
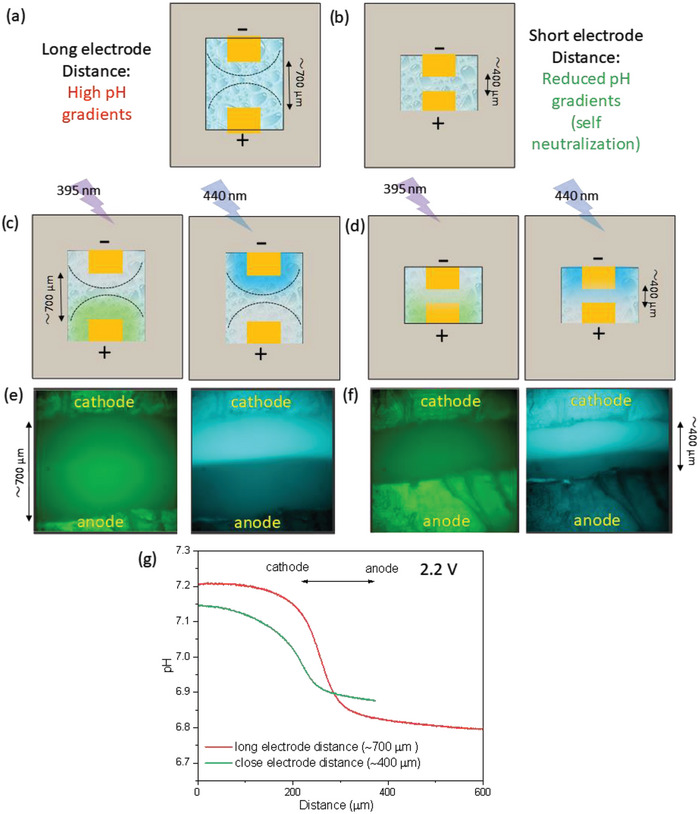
Schematics of the opto‐electrochemical cell at a) long and b) short inter‐electrodes distances without HPTS probe. Schematics demonstrating the fluorescence imaging of the opto‐electrochemical cell at c) long and d) short inter‐electrode distances with HPTS probe at excitation wavelengths of 395 and 440 nm. Fluorescence imaging of the opto‐electrochemical cell at e) long (≈700 µm) and f) short (≈400 µm) inter‐electrode distances with HPTS probe at excitation wavelengths of 395 and 440 nm. Ratiometric analysis demonstrating g) pH profile around the electrodes for short and long inter‐electrode distances.

**Figure 6:**
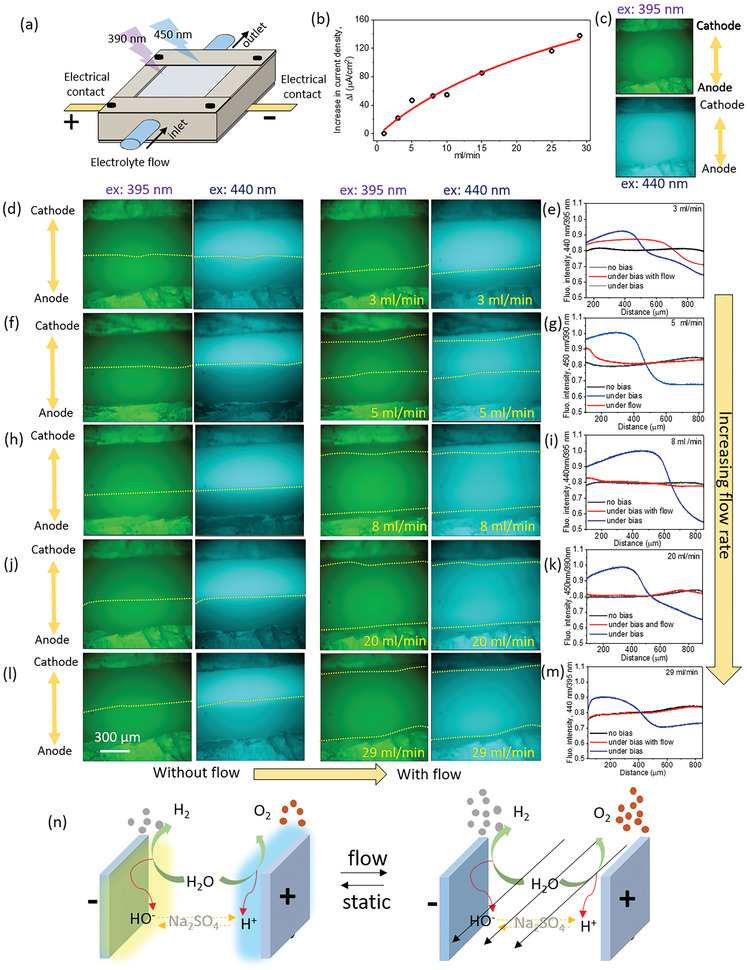
a) Schematics of the opto‐electrochemical flow cell for visualizing electrochemical water splitting processes under controlled flow conditions. b) Increase in current density upon increasing the electrolyte flow rate. c) Fluorescence imaging of the opto‐electrochemical cell under buffered electrolytic conditions (0.25 m PBS). Fluorescence imagining at 395 and 440 nm excitation wavelengths, and corresponding ratiometric analysis of the opto‐electrochemical cell under static and electrolyte flow rates of d,e) 3 mL min^−1^, f,g) 5 mL min^−1^, h,i) 8 mL min^−1^, j,k) 20 mL min^−1^ and l,m) 29 mL min^−1^. n) Schematic demonstrating the mechanism for flow‐induced enhancement in electrocatalytic water splitting under unbuffered neutral electrolytic conditions.

**Figure 7:**
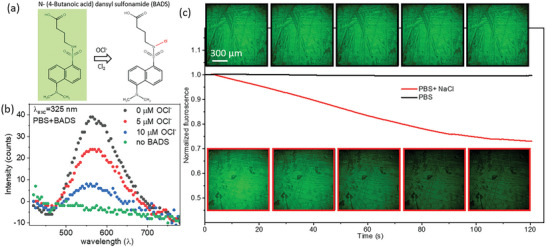
a) Chemical structure of N‐(4‐butanoic acid) dansylsulfonamide (BADS) as a fluorescent probe for OCl^−^/Cl_2_ detection and corresponding quenching of fluorescence upon interaction with OCl^−^/Cl_2_ moiety. b) Fluorescence quenching of 5 µm BADS (at 325 nm excitation wavelength) solution in 0.25 molar phosphate buffer (pH 7) using different concentration of OCl^−^. c) Real‐time spatiotemporal imaging of chloride oxidation during artificial sea water electrolysis (at pH 7) at the electrode surface.

It is now well known that the in‐depth understanding of electrochemical processes is one of the most promising avenues for the achievement of major advancements in different emerging fields for future development. In conjugation, in this work, we have developed opto‐electrochemical technique that can be explored universally for visualizing different electrochemical reactions for unforeseen applications. One such example of major interest is the electrochemical water‐splitting process, for the production of pure hydrogen and oxygen gases. In the past, few literature reports have demonstrated the “optical” visualization of electrochemical cells for in situ imaging of bubbles in electrocatalytic reactions using a high‐speed camera.^[^ ^42^, ^43^, ^44^ ^]^ Zhang et al. performed the micro‐scale investigation of electrochemical cells and provided insights on reaction interface optimization and mass transport optimization by visualizing the gas bubbles during the water‐splitting reaction.^[^ ^45^, ^46^, ^47^ ^]^ Despite these research efforts and advancements in the field during the past decades, scarce efforts have been made toward the chemical moiety‐specific visualization/imaging of the water‐splitting electrochemical reactions. It is to be realized that in this work fluorescence imagining of the electrochemical cell has been performed which gives insights about the chemical changes in the electrochemical system rather than relying on the “optical visualization” which is blind to any chemical changes in the system. As a consequence, in this work, a universal approach for the in situ spatiotemporal visualization of water splitting reaction under neutral water as well as neutral seawater conditions is performed (**Figure** **1**). These two systems are specifically chosen to demonstrate the universality of the technique developed by showcasing the potential applicability toward pH‐based visualization under neutral water (**Figures** **2**, **3**, **4**) along with non–pH change‐based, specific by‐product visualization of chloride oxidation process (Figure 7) in neutral sea water electrolysis process. Furthermore, the influence of electrode distance (**Figure** **5**) and electrolyte flow rates (**Figure** **6**) on the water‐splitting performance is analyzed visually, and directly related to the electrochemical performance of the electrolyzer. The universality of the technique developed can be further explored for the in‐depth understanding of any electrochemical reactions including the highly anticipated intermediate chemical oxygen species influencing the well‐known oxygen evolution/reduction reaction as an example.^[^ ^48^ ^]^

As shown in the schematic, Figure 1a, the operation of a water‐splitting cell in nonbuffered neutral electrolytic conditions leads to a pH < 7 in the OER side and the pH > 7 in the HER side, i.e. the HER side has an alkaline local environment, and the OER side has an acid local environment. This is because, during water splitting under neutral unbuffered conditions, a water molecule is oxidized and reduced to O_2_ and H_2_ with the concomitant formation of H^+^ and OH^−^ at the anode and cathode respectively.^[^ ^49^, ^50^ ^]^ With time, the concentration of H^+^ and OH^−^ ions at the anode and cathode increases leading to the formation of local acidic and basic environments around the anode and cathode respectively. This is usually considered detrimental for electrochemical water splitting as the OER and HER are highly sluggish in these in situ generated acidic and basic environments, however herein, we have explored this phenomenon as a benefit to perform direct spatiotemporal dynamic in situ optical visualization of on‐catalyst water splitting processes. For this purpose, we have explored the pH‐sensitive fluorescent probe HPTS (Figure 2), directly as a homogeneous additional component in the electrolyte medium. and successfully visualized the electrochemical water‐splitting reactions. The fluorescent probe explored in this work is HPTS (8‐hydroxypyrene‐1,3,6‐trisulfonicacid) which belongs to the pyrene family. HPTS is a class of molecule called photoacid, which displays a change in its pKa (≈7.4–≈0.4) thus, an acid‐base transition between their ground and excited state.^[^ ^39^ ^]^ The protonated and deprotonated forms of the HPTS molecule are shown in Figure 2a,b respectively. It is to be emphasized here that the protonated and the deprotonated forms of the HPTS molecule absorb at different wavelengths of ≈400 and ≈450 nm, respectively, thus allowing a ratiometric fluorescence analysis for accurately quantifying the pH changes occurring in a system, by analyzing the fluorescence at different excitation wavelengths. This forms the basis of choosing HPTS molecule as the fluorescent probe to visualize water splitting. The fluorescence spectrum (emission wavelength: 513 nm) of HPTS solution at neutral conditions (pH 7) shows two bands at ≈400 and ≈450 nm, corresponding to the protonated and deprotonated forms, respectively (Figure 2c).

Upon increasing or decreasing the pH, an increase or decrease in the band intensity is observed at ≈450 and ≈400 nm, respectively. Specifically, upon increasing the pH from 7, an increase in the band intensity at ≈450 nm is observed with a concomitant decrease in that at ≈400 nm. Similarly, upon decreasing the pH from 7, an increase in the band intensity at ≈400 nm is observed with a concomitant decrease in that at ≈450 nm. This optical property can be directly explored for pH sensing, and thus fluorescence imaging as a systematic ratiometric trend in the fluorescence ratio for excitation at 440:395 nm is observed upon increasing the pH (Figure 2d). It is very important to notice that a sudden change in the fluorescence intensity ratio (ratiometric response) occurs upon any small deviation from neutralization (pH 7), and thus can be extremely sensitive for visualizing any minute pH changes inside the electrochemical cell upon operation.

Due to the exceptional fluorescence properties of HPTS molecule, it was explored as a fluorescent probe that can be directly added to the electrolyte medium. However, at this point it is very important to analyze the electrochemical properties of the HPTS molecule to investigate its applicability in the electrochemical water splitting system. For this purpose, cyclic voltammetry was performed using a 0.25 m Na_2_SO_4_ electrolyte, with and without the addition of 50 µm HPTS probe, at a low scan rate of 5 mV sec^−1^. Interestingly, it was observed that no oxidation or reduction peaks are observed, with an insignificant change in the cyclic voltammogram, in comparison to that for pristine electrolyte (Figure S1, Supporting Information). This certainly qualifies the use of HPTS as a fluorescent probe in the opto‐electrochemical water splitting cell by direct addition in the electrolyte medium. As the HPTS molecule is demonstrated to be ′electrochemically‐inert″ in the voltage‐window of interest, any change in the fluorescence properties of the electrochemical cell with HPTS as the fluorescent probe can be directly related to the changes occurring due electrochemical production of hydrogen and oxygen. Hence, an electrochemical cell setup was assembled, with two electrodes at a specific distance, and with a transparent optical window that allows real‐time fluorescence microscopy analysis. During electrochemical water splitting reaction under ‘unbuffered’ neutral conditions, upon application of a voltage greater than the onset potential, an increase/decrease in the pH is expected to occur at the cathode/anode compartments, respectively. However, without the addition of HPTS in the electrolyte, the change in pH cannot be visualized/analyzed upon the application of voltage (Figure 3 a,b). On the contrary, the addition of HPTS as a fluorescent probe allows the detection of any change in the environment's pH and thus allows for the in situ spatiotemporal visualization of the individual electrochemical water splitting reactions occurring at the cathode and anode, as well as their influence on the electrolyte bulk at any point inside the cell's space. As HPTS displays two excitation wavelengths, specific for its protonated and deprotonated states (Figure 2c), the opto‐electrochemical cell is exposed to 395 and 440 nm excitation wavelengths for analyzing any resulting deviations in the local environment's pH upon cell operation. Using 395 nm as the excitation wavelength shall display an increase/decrease in the fluorescence intensity at the vicinity of the anode/cathode due to an increase/decrease in the concentration of the protonated form of HPTS upon cell operation (Figure 3c,d). Similarly, excitation with a wavelength of 440 nm shall demonstrate an increase/decrease in the fluorescence intensity at the vicinity of the cathode/anode due to an increase/decrease in the concentration of the unprotonated form of HPTS upon cell operation (Figure 3e,f). Thus, for the overall visualization of the electrochemical water splitting process, we performed a ratiometric image analysis in order to obtain a reliable and accurate pH visualization, with quantification of any known/unknown modulation in the electrochemical reaction.

Based on the proposed hypothesis an opto‐electrochemical cell was fabricated to perform the in situ spatiotemporal dynamic optical visualization of on‐catalyst water splitting processes. Figure 4a shows the schematics of the opto‐electrochemical cell prototype developed and used in this study. The electrochemical cell consists of electrical contacts for the electrodes along with an optically transparent window. Also, electrolyte inlet and outlet ports are included for flowing the electrolyte inside the cell at controlled flow rates. A V‐shaped inlet is provided in the cell geometry to minimize electrolyte flow turbulences and allow for smooth electrolyte flow. The distance of the electrodes' surfaces from the optical window was minimized, keeping in mind the focal distance of the optical objective lens in use. Thus, an opto‐electrochemical cell with an electrode‐to‐optical window distance of 5 mm was fabricated as shown in the digital photograph shown in Figure 4b. The distance between the two electrodes is 5 mm. A large distance between the two electrodes is chosen specifically to minimize the influence of local environment change on the cathode due to that of the anode and vice versa. For that reason, the cathode and the anode are visualized separately. The opto‐electrochemical cell was filled with 0.25 m Na_2_SO_4_ electrolyte along with 50 µm of the HPTS fluorescent probe. The fluorescence imaging of the opto‐electrochemical cell was performed using confocal microscopy under 395 and 440 nm excitation wavelengths. The fluorescence measurements were performed at a fixed voltage of 2.4 V, and the space volumes around the cathode (Figure 4c) and anode (Figure 4d) are visualized in real‐time. Before the application of any voltage, the electrolyte around the electrodes (both cathode and anode) does not show any pH changes, and thus no optical changes are observed, and exhibit a 440:395 nm emission ratio of 1.2 (Figure 4c) at the surface of the electrodes, as well as far from the surface of the electrodes (toward bulk electrolyte). As soon as the mentioned voltage is applied, due to the formation of acidic (H^+^) and basic (OH^−^) by‐products around the cathode and the anode, respectively, a rapid change in the fluorescence intensity is observed. At the close vicinity of the cathode, a decrease/increase in the fluorescence intensity for excitation at 395/440 nm is observed, due to the formation of alkaline conditions, leading to an increase in the deprotonated form of the HPTS probe. As a result, an increase in the 440:395 nm fluorescence ratio near the cathode is observed, with no changes observed away from the cathode's surface at 5 s after voltage operation. As the time for voltage operation is increased, an increase in the 440:395 nm fluorescence ratio is observed (1.62 at 20 s), along with an increase in the space volume, or front width, where the effect of alkalinity is observed due to diffusion of OH^−^ species toward the bulk electrolyte from the cathode's surface. Similarly, the influence of acidification at the anode is observed, with the formation of a moving acidic front with increased voltage operation times, due to the diffusion of H^+^ species from the electrode toward the bulk (Figure 4d). As a result, with increasing voltage application time, a decrease in the 440:395 nm fluorescence ratio is observed from 1.2 at 0 s, 1.05 at 5 s, 0.96 at 20 s, and 0.66 at 100 s. The fluorescence ratios were further analyzed and calibrated to finally obtain the change in the pH around the electrodes' surface, and the in situ spatiotemporal evolution of electrolytic medium under water splitting conditions (Figure 4e,f). As expected, as soon as the potential is applied, an increase in the pH is observed around the cathode surface which further extends up to a distance of 400 µm at 5 s (Figure 4e). The pH further increases and saturates at ≈pH 8 within 100 s and extends to more than 1 mm distance from the electrode's surface, which was beyond the field of view of the objective lens. Similarly, a decrease in the pH was observed around the vicinity of the anode, which increases temporally, reaching a value of ≈pH 6 at 100 s (Figure 4f). As expected, the pH changes are at their maximum level in close vicinity of the electrodes' surface, while the bulk electrolyte exhibits lesser deviation from neutrality, which is expected due to diffusion limitations. Thus, for the first time, direct in situ spatiotemporal imaging of the chemical phenomena happening during water splitting processes is performed and is in correlation with the previous literature knowledge. As a result, a reliable imagining technique for direct visualization of electrochemical water splitting reactions is successfully developed here.

The fluorescence‐based imaging technique developed in this study was applied for a better understanding of the water‐splitting electrochemical processes. The detrimental effect of the rapid and dramatic pH changes at the electrode surface is due to the poor OER and HER under acidic and basic conditions, respectively. At large inter‐electrodes distances, the large increase or decrease in the pH at the electrodes' surroundings will have a significant detrimental effect on the overall water‐splitting performance. However, the point of interest is if the electrodes are close enough, at certain inter‐electrodes distances, that the acidic and basic fronts developing at the anode and cathode can actually ′self‐neutralize″ each other, leading to enhanced electrochemical behavior as described in Figure 5a,b. We here demonstrated, visually in Figure 4, that the water splitting under neutral nonbuffered conditions causes a rapid and sharp increase in the acidity/alkalinity at anode/cathode surfaces, however, the influence of distance between the electrodes is yet to be studied. For this purpose, two opto‐electrochemical cell configurations were investigated, with different inter‐electrode distances of ≈700 µm (far) and ≈400 µm (close), and the in situ spatiotemporal visualization for the comparative resulting pH changes was performed under excitation wavelengths of 395 and 440 nm (Figure 5c,d). Fluorescence spatiotemporal imaging shows that in both cases, far and close inter‐electrodes distances, an increase/decrease in the fluorescence intensity around the electrodes is observed due to the formation of acidic/basic fronts at excitation wavelengths of 395 and 440 nm. Interestingly, a clear difference in the electrolyte media characteristics around the cathode and the anode is observed with the bright and dark fluorescent phases merging at the center point between the electrodes. These dark/bright phases are due to the formation of protonated and deprotonated forms of HPTS at the anode and cathode, respectively (Figure 5e,f). To understand the effect of the decrease in the distance between the electrodes, the fluorescence images were ratiometrically analyzed in‐depth to see the comparative pH changes occurring at the electrodes at far and close inter‐electrode distances (Figure 5g). The pH profile along the whole distance between the electrodes is plotted for short (≈400 µm) and far distance (≈700 µm) between the electrodes. The pH for both far and close inter‐electrode distances demonstrates a stable pH at close vicinity to the cathode surface, then decreases sharply toward the center point between the two electrodes and finally stabilizes close to the anode. This is expected to be due to the formation of acidic and basic environment spaces around the anode and cathode, respectively, during the electrochemical water splitting process. Interestingly, a lesser deviation in the pH is observed from cathode to anode at close electrode inter‐distances. Precisely, a pH change of, ΔpH: 0.41 is observed for electrodes at far distances in comparison to that of, ΔpH:0.26 for electrodes at close distances which clearly shows that a much higher (57% increase in ΔpH) deviation in the pH is observed when the cathode and anode are far, in comparison to when they are close to each other. These results clearly indicate toward a lesser deviation from neutrality when the electrodes are close to each other, due to a self‐neutralization phenomenon, in comparison to the case when the electrodes are far from each other. Thus, the direct spatiotemporal visualization of water splitting at different inter‐electrode distances provides a new practical direction to the enhanced non‐buffered neutral water splitting, where the detrimental acidic or basic environment formation at the vicinity of the electrode can be effectively decreased or totally eliminated by keeping the electrodes at a certain close distance in order to attain self‐neutralization, thus enhanced water splitting performances.

The detrimental effect of pH changes at the electrodes during water splitting under neutral conditions can also be minimized by replacing the water “shell” around the electrodes continuously. One plausible practical approach to do so is by flowing electrolyte in a flow‐type electrolysis cell, which can provide in situ ′buffering″ conditions. The in situ spatiotemporal imaging technique developed in this study was explored to visualize the water‐splitting reactions under flow conditions. For this purpose, the opto‐electrochemical cell developed in this work was equipped with flowing electrolyte capabilities, through the use of an external automated syringe pump (Figure 6a). To analyze the effect of different flow conditions on the electrochemical water splitting process, the change in the measured electrolyzer's current under different electrolyte flow rates was investigated under static and flow conditions (Figure 6b). As expected, an increase in the electrolyzer's current density was observed under flow conditions, in comparison to that observed under static electrolyte conditions. Interestingly, an increase in the electrolyzer's current density was also observed with increasing electrolyte flow rates. The observed increase in the current density, (Δ*I*) rises rapidly at low flow rates and then tends to stabilize at a certain plateau at increasing flow rates. To understand the increase in the electrolyzer's current density at increasing flow rates, the in situ visualization of the electrochemical water splitting cell under operation was performed under no‐flow and under different flow rate conditions. As a control, the in situ visualization of an electrolyzer cell with ′buffering″ electrolyte was performed under no‐flow static conditions. As expected, no change in the spatiotemporal fluorescence imaging results was observed upon voltage application in the ‘buffered’ cell, as the corresponding acid and base by‐products formed at the anode and cathode, respectively, are immediately and completely compensated by the externally used buffering agents in this control cell (Figure 6c). However, in a nonbuffered neutral medium, a clear spatiotemporal distinction in the fluorescence intensity, at the cathode and the anode electrodes, for both 395 and 440 nm is observed due to the formation of basic and acidic space regions. To analyze the effect of flow on these acidic and basic conditions, the in situ fluorescence imaging was performed under static and flow conditions at different flow rates (Figure 6d,f,h,j,l). Furthermore, the spatiotemporal ratiometric analysis was performed to visualize any changes occurring upon voltage application, and the concomitant ′neutralization″ effect happening upon flowing the electrolyte (Figure 6e,g,i,k,m). Interestingly, under a very slow electrolytic flow condition of 3 ml min^−1^ (total cell exchange volume factor of 6 exchange per min), a considerable ′shrinking″ in the acidic and basic space regions is observed, however, it can be observed that the acidic and basic space fronts are still clearly visible under these flow conditions (Figure 6d). The ratiometric analysis further demonstrates a clear spatiotemporal pH change upon the application of voltage (static condition, blue color) in comparison to the nonbias condition (black color). Upon flowing the electrolyte under a voltage bias, a lesser deviation in the fluorescence intensity ratio (440:395 nm) is observed (red color) in comparison to that without flow (Figure 6e). Upon increasing the electrolyte flow rate, a systematic decrease in the acidic and basic space fronts is observed, with the nonbias conditions slowly matching the results observed for flow conditions upon voltage bias application. Specifically, at a high flow rate of 29 mL min^−1^ (total cell exchange volume factor of 58 exchange per min), no acidic or basic space fronts are visible in the spatiotemporal fluorescence imaging, with the nonbias condition almost perfectly matching with that of bias application at high flow condition in the fluorescence intensity ratio plot (Figure 6i,m). Thus, the plot for the increase in current density with the increase in flow rates can be directly related to the reduction, or complete inhibition, of the detrimental acidic and basic space fronts forming at the anode and cathode, respectively, upon electrolytic flow during the water splitting process. At low flow rate conditions, though the electrolyte is replaced at the electrode's surface, however, the electrolyte replacement rate is not sufficient to totally eliminate the dramatic spatiotemporal pH changes happening at the electrodes' surface, leading to a significant increase in the electrolyzer's current density. As the electrolyte flow rate increases, a systematic decrease in the electrolyzer's current density is observed, due to increased electrolyte replacement rate. At higher flow rates, the curve of the electrolyzer's increase in current density reaches a plateau, because at these high flow rates an effectively fast electrolyte replacement rate is attained, thus effectively inhibiting the formation of the detrimental acidic and basic space fronts at the vicinity of the electrodes during the water splitting process.

The corresponding pH shifts around the electrodes under neutral unbuffered electrolytic conditions are expected due to the mass transport limitations of the ions which is well understood based on the Nernst plank equation:

(1) $$N_{i}=-D_{i}\nabla c_{i}+\frac{-z_{i}F}{RT}D_{i}c_{i}\nabla \emptyset_{l}+c_{i}\gamma$$

where, *N_i_* represents the flux vector, *D_i_* represents the diffusion coefficient, *c_i_* is the concentration and *z_i_* is the charge of any specie *i* in the electrochemical system. Further, the ∅_*l*_, γ, R, F and T represents the local electrolyte potential, electrolyte velocity vector, gas constant, Faraday constant and temperature respectively. As observed in Equation (1), the mass transport flux of chemical species in the electrolyte is governed by 1) diffusion due to concentration gradient, 2) migration of ions due to the applied voltage and 3) the convection in the electrolyte solution. Thus, the pH gradient during electrochemical water splitting under neutral conditions can be reduced by increasing any of these contributions. In this work, diffusion and migration are kept constant as the electrocatalytic performance is visualized and analyzed under nonbuffered, same electrolytic conditions and fixed applied voltage respectively. However, as a step toward decreasing the detrimental formation of acidic and basic components, the third contribution i.e. the convection in the system is intentionally increased by flowing electrolyte to replace the developing acidic and basic fronts around the anode and cathode respectively by fresh electrolyte. This obviously will increase the flux of chemical species as per Equation (1), concomitantly will also majorly inhibit the detrimental effect of pH changes on the electrocatalytic activity of any electrolyte for OER and HER in the neutral medium in comparison to that in acidic and basic medium respectively (Figure 6m). Further, the rate of transfer of charge of the H^+^ and OH^−^ ions via the electrolyte (micro seconds for strong electrolyte) is orders of magnitude higher than the flow rates of the electrolyte, and thus, the charge transport has insignificant variance with the applied flow. Overall, the flow of electrolyte acts as a route to replace the acidic and basic fronts around the anode and the cathode, thereby increasing the flux of chemical species and providing an optimum electrolytic environment without the use of buffers and thus is evident as an enhancement in the current density with increasing flow rates. Similar results are published in the recent past by our group where extensive research on the effect of flow on the electrocatalytic performance is observed and an unprecedented cell voltage of 1.39 V at 10 mA cm^−2^ is observed for direct sea water splitting under thermo‐hydrodynamic conditions.^[^ ^11^ ^]^

Inspired by the unprecedented application of imaging in situ electrochemical reaction technique developed in this work, and to analyze the universality of the technique developed, we extended the work to visualize significantly different system by using a different fluorescent probe for detecting a specific product in the reaction rather than detection the pH change. As an example, the visualization of chloride oxidation at the anode during direct sea water splitting is temporally visualized using a dansylsulphoamide based fluorescent probe, N‐(4‐butanoic acid) dansylsulfonamide (BADS)^[^ ^51^ ^]^ directly in the electrolyte during electrolysis. BADS is an exceptionally sensitive and selective fluorescent molecule which quenches upon interaction with OCl^−^/Cl_2_ moieties even at very low concentrations as shown in **Figure** **7**a. The optical properties and sensitivity toward OCl^−^/Cl_2_ moieties was analyzed by testing the excitation spectra of BADS with different concentration of hypochlorite ion (OCl^−^). It is important to realize that as observed in the previous sections water splitting under neutral conditions is prone to pH changes around the electrode and BADS is sensitive to pH changes, all the analysis was further performed in buffered neutral conditions to avoid any changes occurring due to the change in pH. The emission spectra of 5 µm BADS at an excitation wavelength of *λ*_exc _= 325 nm show a clear emission band centered at ≈560 nm. Interestingly, even with the addition of 5 µm OCl^−^, a significant decrease in the fluorescence intensity is observed which decreases further on increasing the OCl^−^ concentration to 10 µm as shown in Figure 7b. Thus, the ability of BADS to detect small concentrations of OCl^−^ under neutral conditions is demonstrated and can be further applied for imaging the in situ oxidation of Cl^−^ during sea water splitting. For the purpose, BADS was directly used as a fluorescent probe in the opto‐electrochemical imaging cell developed in this study for imaging chloride oxidation during sea water splitting. The BADS based opto‐electrochemical cell with 0.5 m NaCl (artificial seawater) in buffered neutral electrolyte with Pt electrodes was operated at 3 V to ensure the oxidation of Cl^−^ to form OCl^−^/Cl_2_ moieties at the anode. Hence, the spatiotemporal imaging of the anode was performed at *λ*_exc _= 395 nm at chronoamperometric conditions of 3 V in electrolyte with and without 0.5 m NaCl (Figure 7c). The BADS in the electrolytic system demonstrates initial intrinsic fluorescence at the electrode surface. Remarkably, upon application of the external voltage, a decrease in the fluorescence intensity was observed due to the oxidation of Cl^−^ to OCl^−^/Cl_2,_ which thereby quenches the intrinsic fluorescence of BADS. Interestingly, a systematic decrease in the overall fluorescence intensity was observed with increasing time because of the systematic increase in the OCl^−^/Cl_2_ concentration in the opto‐electrochemical cell during electrolysis. Further, an insignificant change in the fluorescence intensity was observed upon electrolysis of PBS without NaCl, thus confirming the systematic decrease demonstrated in the in situ visualization to the systematic oxidation of chloride ions which eventually quenches the fluorescence. As a result, the opto‐electrochemical imaging technique developed in this work was successfully extended to a significantly different (pH independent) electrochemical reaction using a totally different fluorophore. As another example, hydroethidine‐based fluorophores can be explored for visualizing and probing in‐depth insights on the reactive oxygen intermediates which are known to influence the major energy‐based electrochemical reactions including the oxygen reduction and oxygen evolution reactions.^[^ ^52^, ^53^, ^54^ ^]^ Water splitting is usually carried out under alkaline conditions to catalyze the sluggish oxygen evolution reaction. However, due to the highly corrosive condition under extreme pH, there is a continuous quest to perform the water‐splitting reaction in neutral conditions. Under neutral unbuffered electrolytic conditions, significant pH changes are expected at the electrodes which are visualized in this work using an HPTS‐based fluorescent probe as an example as a step forward toward the visualization of any chemical reaction. The formation of these acidic and basic fronts will not be as significant under highly concentrated alkaline conditions or highly concentrated acidic conditions. However, under, low‐concentration alkaline/acidic conditions, this approach can be directly applied to observe the pH variation around the electrodes. Given the universality of this approach, any fluorescent probe that is stable and responsive under alkaline conditions can be directly added to the electrolyte to visualize water splitting under alkaline conditions. Recently, various fluorophores are established to demonstrate a stable and repeatable response to pH changes under highly acidic (imidazole and Xanthene based fluorophores)^[^ ^25^, ^26^ ^]^ and alkaline conditions (perylene tetra‐(alkoxycarbonyl, boron–dipyrromethene based fluorophores)^[^ ^27^, ^28^, ^29^, ^30^ ^]^ which can be directly employed in this approach to visualize water splitting under highly acidic or alkaline conditions.

Thus, the spatiotemporal imaging approach developed in this work has been shown to be able to universally visualize in real‐time, as never seen before, the on‐electrodes electrochemical processes in the electrolyte itself. Importantly, the applicability of this proposed approach was successfully evaluated for practically realizing the ′self‐neutralization″ phenomenon via decreasing the inter‐electrodes distance, and also for understanding the enhancing effects of electrolyte's flow on the overall electrochemical water splitting reaction performance. Furthermore, the use of OCl^−^/Cl_2_ specific probes was successfully explored for imaging in situ electro‐oxidation of Cl^−^ in sea water oxidation, remarkably justifying the universality of the technique developed for temporally visualizing any electrochemical reaction for in‐depth unprecedented.

## 3 Conclusion

In conclusion, a novel experimental paradigm for the in situ spatiotemporal ′visualization″ of electrochemical reactions, with water splitting applied as model system, is developed in this study. For this purpose, the photoacid molecule HPTS (8‐hydroxypyrene‐1,3,6‐trisulfonicacid) is explored as a fluorescent probe for monitoring any spatiotemporal pH deviations occurring in the electrochemical system. The applicability of the opto‐electrochemical platform developed in this work was successfully evaluated in the real‐time spatiotemporal analysis of the electrocatalytic water splitting reactions occurring under neutral electrolytic conditions. As a result, for the first time, the direct spatiotemporal dynamic in situ optical visualization of on‐catalyst water splitting processes is performed by adding a single fluorescent pH‐probe directly into the electrolytic medium. Interestingly, the clear developing temporal formation of acidic and basic space environments around the anode and cathode, respectively, is observed, both concomitantly exhibiting a temporal increase in dimensions as a consequence of the enhanced accumulation and diffusion of these formed acid and base by‐products. The opto‐electrochemical cell developed in this work was further explored to investigate and analyze for the first time the water splitting processes at neutral conditions. The in situ spatiotemporal visualization of the water splitting processes at small inter‐electrode distances was successfully explored to unprecedentedly realize the possibility of ′self‐neutralization″ of the detrimental acidic and basic space fronts, at these small distances. Furthermore, the enhancement in the electrocatalytic water splitting activity under controlled flowing electrolyte conditions was thoroughly examined by the direct spatiotemporal visualization approach. It was clearly observed that at increasing electrolyte flow rates, a considerable decrease in the dimensions of the acidic and basic space fronts, formed during the water splitting process, is observed, thus decreasing, and finally completely inhibiting, the detrimental effects of the sharp pH changes occurring on the electrodes' vicinity during the performance of water splitting under neutral conditions. Furthermore, to demonstrate the universal extension of the experimental tool developed in this work to more specific reaction by products, direct in situ visualization of OCl^−^/Cl_2_ produced during electrolysis of sea water was successfully performed using N‐(4‐butanoic acid) dansylsulfonamide as a selective fluorophore. Thus, a unique opto‐electrochemical spatiotemporal real‐time universal visualization approach was successfully developed and demonstrated in this work, which holds the potential to open new fields of research and provide new impactful insights to the field of electrochemistry and its related applications.

## 4 Experimental Section

### Opto‐Electrochemical Cell

To perform in situ visualization of electrochemical measurements, the opto‐electrochemical cell was fabricated with an optical glass window on its top face. Precleaned nickel foil (99.99%) and platinum foil (99.99%) sheets were used as the electrode materials for the opto‐electrochemical investigations. The distance between the optical glass window of the electrochemical cell and the electrodes' surface being visualized is ≈5 mm. This small distance of ≈5 mm is important as the cell is being visualized in an inverted fluorescence microscope (details given in the later section of the Experimental Section) having an intrinsic small working distance (distance between the glass window and the objective lens) of the objective lens (10×) used in this study. Furthermore, in order to perform electrolyte flow measurements, the cell was equipped with inlet and outlet ports connected to a syringe pump with an adjustable flow rate. The flow rates during the electrochemical measurements were modulated from 3 to 29 mL min^−1^.

### Electrolytes and Fluorophores

The electrolyte used in this work is 0.25 m Na_2_SO_4_ with the co‐addition of 10 µm HPTS (8‐hydroxypyrene‐1,3,6‐trisulfonicacid) (Sigma, 6358‐69‐6). Dilute potassium hydroxide and dilute hydrochloric acid were used to make solutions with different pH, using an Oakton pH analyzer. As a control, 0.25 m phosphate buffer, with the co‐addition of 10 µm HPTS, was also tested. Further, the opto‐electrochemical detection of chloride oxidation was performed using 50 µm N‐(4‐butanoic acid) dansylsulfonamide (BADS) fluorophore by directly added in the electrolyte (0.5 m NaCl in phosphate buffer). N‐(4‐butanoic acid) dansylsulfonamide 99%, CAS: 102783‐77‐7 was purchased Henan Bao Enluo Trading Co. ltd, China.

### Fluorescence Based Visualization

The operational opto‐electrochemical imaging studies were performed using an inverted fluorescence Thunder microscope (LEICA MD4000M Fluorescence microscope with LEICA DFC450 camera). The pH‐based opto‐electrochemical visualization was performed using ratiometric fluorescence analysis using HPTS as a fluorescent probe at excitation wavelengths of 395 and 440 nm. The visualization of chlorine‐based by‐products was performed at an excitation wavelength of 395 nm using BADS as the selective fluorescent probe. The opto‐electrochemical fluorescence images and movies were analyzed using the Leica image analysis software (LAS X). Before the fluorescence imagining, the concentration and the excitation wavelengths of the respective fluorophore were optimized using a microplate well reader (TECAN Infinite 200, NEOTEC Scientific Instruments Ltd).

### Electrochemical Measurements

All the electrochemical measurements were performed in a two‐electrode configuration using PalmSens electrochemical instrument in opto‐electrochemcial cell under a fluorescence microscope.

### Application of Visualizing Water Splitting

The opto‐electrochemical imaging was also performed at two different electrode distances of 400 and 700 µm to analyze the effect of electrodes' distance on the change in pH between the electrodes as a preliminary step toward additive free water splitting under small electrode distances. Toward the same line of reducing the pH variations at the electrodes, the in situ visualization of the opto‐electrochemical cell was performed under varying electrolyte flow rates from 3 to 29 mL min^−1^ as a step toward visualizing the mechanism of mechanical buffering the water splitting reactions under nonbuffered neutral electrolytic conditions.

## Conflict of Interest

The authors declare no conflict of interest.

## Supporting information

Supporting Information

## Data Availability

The data that support the findings of this study are available from the corresponding author upon reasonable request.
