# Supplementary material for: Universal Approach to Direct Spatiotemporal Dynamic In Situ Optical Visualization of On‐Catalyst Water Splitting Electrochemical Processes
Source: Adv Sci (Weinh). 2024 Apr 22;11(24):2401258. doi: 10.1002/advs.202401258 (PMC11199991; doi:10.1002/advs.202401258)
Supplement: Supplementary file 1 — Supporting Information [file ADVS-11-2401258-s001.pdf]

ADVANCED SCIENCE
Open Access

Supporting Information

for *Adv. Sci.*, DOI 10.1002/advs.202401258

Universal Approach to Direct Spatiotemporal Dynamic In Situ Optical Visualization of On-Catalyst Water Splitting Electrochemical Processes

Gaurav Bahuguna and Fernando Patolsky**

Supporting Information

Universal Approach to Direct Spatiotemporal Dynamic *in-situ* Optical Visualization of On-Catalyst Water Splitting Electrochemical Processes

Gaurav Bahuguna[1*], Fernando Patolsky[1,2*]

[1]*School of Chemistry, Faculty of Exact Sciences, Tel Aviv University, Tel Aviv, 69978, Israel.*

[2]*Department of Materials Science and Engineering, the Iby and Aladar Fleischman Faculty of Engineering, Tel Aviv University, Tel Aviv 69978, Israel.*

[*]*Corresponding Authors*:

Prof. Fernando Patolsky (*fernando@post.tau.ac.il*)

Dr. Gaurav Bahuguna (*gauravb@tauex.tau.ac.il, bahuguna.1@iitj.ac.in*)

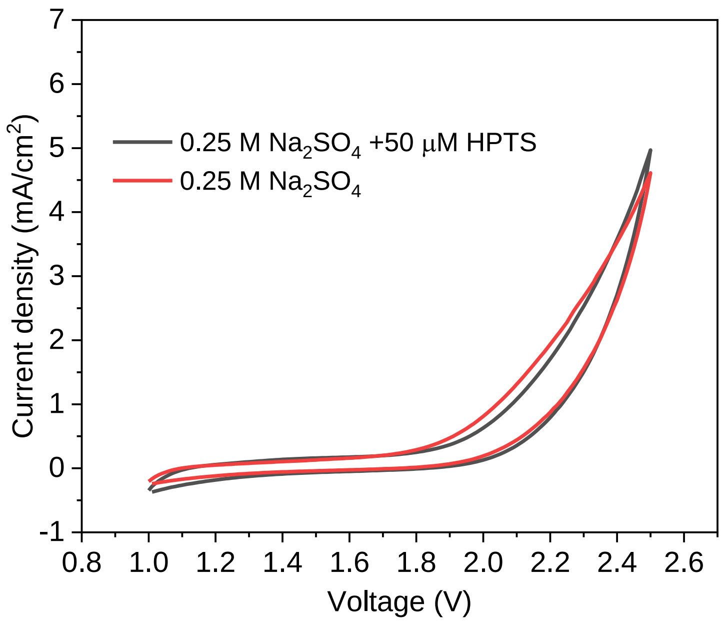

Figure S1: Cyclic Voltammogram of 0.25 M Na_2SO_4 and 50 µM HPTS in 0.25 M Na_2SO_4 at low scan rate of 5 mV/s.
